# Therapeutic potential and safety challenges of antimicrobial peptides and peptidomimetics against ESKAPEE pathogens: a systematic review with quantitative analysis

**DOI:** 10.1093/jac/dkag255

**Published:** 2026-07-27

**Authors:** Elias Shiferaw Mekonen, Shyam Kumar Mishra, Umme Laila Urmi, Yihenew Million Yeshtila, Furqan A Maulvi, Naresh Kumar, Mark D P Willcox

**Affiliations:** School of Optometry and Vision Science, Faculty of Health and Medicine, University of New South Wales, Sydney, NSW 2052, Australia; School of Optometry and Vision Science, Faculty of Health and Medicine, University of New South Wales, Sydney, NSW 2052, Australia; School of Optometry and Vision Science, Faculty of Health and Medicine, University of New South Wales, Sydney, NSW 2052, Australia; Paediatrics and Child Health, School of Clinical Medicine, University of New South Wales, Sydney, NSW 2052, Australia; School of Optometry and Vision Science, Faculty of Health and Medicine, University of New South Wales, Sydney, NSW 2052, Australia; School of Chemistry, University of New South Wales, Sydney, NSW 2052, Australia; School of Optometry and Vision Science, Faculty of Health and Medicine, University of New South Wales, Sydney, NSW 2052, Australia

## Abstract

**Background:**

Antimicrobial peptides and peptidomimetics have emerged as promising alternatives to traditional antibiotics for MDR bacterial infections. Their advancement, however, is often limited by toxicity and poor pharmacokinetics. In this review, we apply a rigorous quantitative framework to evaluate the therapeutic balance of potency and safety for these agents, offering new perspectives that extend beyond previous descriptive analyses.

**Methods:**

We systematically searched key biomedical databases (January 2000 to May 2025) for studies reporting antimicrobial potency, cytotoxicity, selectivity index, stability and *in vivo* efficacy. Bias was assessed using an adapted laboratory animal experimentation quality tool. Data were synthesized using a pooled geometric mean approach to compare therapeutic windows across compound.

**Results:**

Of 136 peptides from 47 studies, natural antimicrobial peptides were most potent *in vitro* against Gram-negative bacteria but frequently caused significant host toxicity. Limited cytotoxicity data prevented robust selectivity analysis for these natural compounds. Peptidomimetics generally provided a wider safety margin than synthetic peptides, though toxicity was context dependent. Major translational barriers, including protein binding and cation effects, diminished *in vivo* efficacy. Notably, some rationally designed compounds achieved therapeutic benefit in animal models without acute toxicity, unlike traditional agents such as polymyxin B.

**Conclusions:**

Advancing antimicrobial peptides and peptidomimetics into clinical use will require overcoming the fundamental trade-off between potency and safety. Natural compounds remain restricted by toxicity, while synthetic agents and peptidomimetics promise better safety but face pharmacokinetic challenges. Future research should prioritize innovative delivery approaches to enhance efficacy and reduce toxicity, enabling these novel therapies to address MDR infections.

## Introduction

Antimicrobial resistance (AMR) continues to accelerate as one of the gravest threats to global public health and sustainable development. The widespread overuse and misuse of antibiotics have accelerated the emergence and dissemination of antimicrobial-resistant bacteria.^1^ In 2019, it was estimated that bacterial AMR was directly responsible for 4.95 million deaths globally, establishing it as a leading cause of global mortality. Projections suggest that, without urgent intervention, AMR could result in up to 10 million deaths annually by 2050, with profound economic ramifications.^2^

In response to this mounting challenge, the WHO and the CDC have identified priority bacterial pathogens urgently requiring novel therapeutic strategies. Chief among these are the so-called ‘ESKAPE’ pathogens, *Enterococcus faecium*, *Staphylococcus aureus*, *Klebsiella pneumoniae*, *Acinetobacter baumannii*, *Pseudomonas aeruginosa* and *Enterobacter* species, which are notorious for their MDR.^3–5^ Given its global clinical significance and its formidable resistance mechanisms, *Escherichia coli* is also included, expanding the group to ‘ESKAPEE’ pathogens.^6,7^ Collectively, these bacteria account for a broader spectrum of infections, ranging from bloodstream and respiratory tract infections to those involving the gastrointestinal, urinary tracts, skin and soft tissue, bone, joint and medical device-associated infections.^7^

Polymyxins, particularly polymyxin B and colistin (polymyxin E), have re-emerged as last-resort agents against carbapenem-resistant Gram-negative bacteria. Their bactericidal effect depends on binding to LPS in the bacterial outer membrane, displacing stabilizing divalent cations (Ca^2+^, Mg^2+^) and disrupting membrane integrity, which culminates in cell lysis and death.^4^ However, the utility of polymyxins is severely constrained by their dose-dependent nephrotoxicity and neurotoxicity; nephrotoxicity occurs in up to 40%–60% of patients.^8,9^

The stagnation in antibiotic discovery galvanized renewed interest in antimicrobial peptides (AMPs) and peptidomimetics as a potential alternative to conventional antibiotics. AMPs are typically short (5 to 100 amino acids), amphipathic molecules with a net positive charge (+2 to +11) and high hydrophobic content (∼50%), which enables their targeting of bacterial membranes.^10^ Similar to polymyxins, these peptides exploit electrostatic interactions with negatively charged surfaces of bacterial membranes. Their broad-spectrum activity, rapid bactericidal effects and relatively low propensity for resistance make them compelling candidates for next-generation antimicrobial development.^11,12^

### Mechanisms of action

AMPs demonstrate broad-spectrum antimicrobial activity through diverse and often synergistic mechanisms, which substantially reduces the risk of resistance selection compared with conventional antibiotics that typically target a singular cellular process.^10,13,14^ The predominant mechanism, analogous to that of polymyxins, involves direct perturbation of bacterial cell membranes. This is achieved through electrostatic attraction between the cationic peptide and the anionic bacterial surface, followed by hydrophobic insertion that disrupts membrane integrity, increases permeability and ultimately causes cell lysis.^10^ In Gram-negative bacteria, this process begins with the binding of the peptide to LPS, facilitating penetration and depolarization of the underlying cytoplasmic membrane.^15,16^ The ability of AMPs to permeate microbial membranes is closely linked to their propensity to adopt specific secondary structures such as α-helix or β-sheet upon contact with lipid bilayers.^17,18^ Beyond membrane disruption, AMPs may interfere with critical intracellular processes, including nucleic acid and protein synthesis, inhibit cell wall biosynthesis, and disrupt cell division.^19^

The functional repertoire of AMPs extends well beyond broad-spectrum antibacterial activity, encompassing anti-biofilm, antifungal, antiviral and even anticancer properties. These effects often exploit selective cytotoxicity directed at microbial or malignant cell membranes.^1,15,20,21^ In the context of biofilm inhibition, antimicrobial peptides disrupt biofilms by several distinct mechanisms: dissipating membrane potential within biofilm-embedded cells, interfering with bacterial communication systems such as quorum sensing, degrading extracellular polymeric substances (including polysaccharides and DNA), inhibiting the stringent response via modulation of (p)ppGpp, and down-regulating genes essential for biofilm maintenance and transport.^14,22^

Furthermore, as integral components of host defence, antimicrobial peptides possess potent immunomodulatory properties. They are capable of tempering excessive inflammatory responses by binding to and neutralizing molecules such as LPS and bacterial exotoxins.^23,24^

### Classification of AMPs

AMPs represent a remarkably diverse class of molecules, broadly categorized as either natural or synthetic. Natural AMPs function as key components of the innate immune system across a wide range of organisms, including vertebrates (e.g. LL-37, BMAP-18), amphibians (brevinins, aureins), marine species (arenicin-1, tachyplesin I), insects (cecropins), arachnids (gomesin), mammals (protegrins) and plants (NCR247). Others may originate from bacteriophages, venoms or toxins, such as scorpion-derived peptides.^10,25,26^ By contrast, synthetic AMPs are produced via chemical synthesis or rational design incorporating strategies such as amino acid substitution, lipidation, cyclization or multimerization to optimize activity, enhance stability and minimize toxicity.^14,21,27,28^ Classification can also be based on secondary structure (α-helical, β-sheet, extended/random coil, cyclic), physicochemical properties (length, charge, hydrophobicity) or biological activity. Peptidomimetics constitute a related class: these are non-natural, synthetic compounds engineered to recapitulate the structural and functional features of natural peptides, with chemical modifications designed to further enhance antimicrobial activity, stability and biocompatibility.^29^

In response to the escalating threat of AMR, both AMPs and peptidomimetics are under active investigation as alternative therapeutics to conventional antibiotics. A critical consideration for clinical translation is a sufficient selectivity index (SI), defined as the ratio of the 50% cytotoxicity concentration (CC_50_) to the MIC, which reflects the therapeutic window. The potent membrane-disrupting action of these peptides, while effective against bacteria, often results in dose-limiting cytotoxicity to eukaryotic cells and rapid degradation by proteases *in vivo*, paralleling the toxicity challenges seen with existing cationic antibiotics like polymyxins. Consequently, the clinical application of AMPs is frequently restricted to localized delivery approaches.^30^

Although several recent reviews, such as those by Mahlapuu *et al.* (in 2020)^31^ and Magana *et al.* (also in 2020),^32^ have addressed the translational challenges and efficacy of antimicrobial peptides, most published literature relies on descriptive aggregation of heterogeneous data. These reviews frequently report broad, overlapping MIC ranges without quantitatively disentangling antibacterial potency from host cell cytotoxicity. As a result, a critical knowledge gap persists regarding systematic quantification of the potency-safety trade-off that determines clinical viability.

To address the critical barriers limiting the therapeutic translation of AMPs and peptidomimetics this review introduces a novel quantitative synthesis approach using a pooled geometric mean framework. By calculating pooled geometric mean MICs and systematically mapping them against corresponding mammalian cytotoxicity data, we establish a comprehensive potency safety landscape. This enables direct comparison of selectivity indices across compound classes targeting ESKAPEE pathogens. Furthermore, by quantifying key physiological stability barriers, including serum protein binding and proteolytic degradation, our analysis delineates which structural classes possess the most favourable therapeutic windows for systemic clinical development.

## Methods

A comprehensive systematic literature search was undertaken to identify, synthesize and critically appraise the scientific literature on the antimicrobial efficacy, host safety, biophysical stability, and *in vivo* safety of AMPs and peptidomimetics. The methodology adhered to established systematic review guidelines to ensure transparency, reproducibility and methodological rigour.

### Search strategy

A systematic literature search was conducted in PubMed, Embase and Scopus. The final search was completed on 15 May 2025, covering studies published from 1 January 2000 to that date. The search strategy combined free-text keywords and controlled vocabulary, including MeSH terms for PubMed term explosions where appropriate. For supplementary and grey literature, Google Scholar was searched using the same primary search strings, with results restricted to English-language publications from 2000 onwards and sorted by relevance. Backwards and forwards citation tracking was performed by screening the reference lists of included studies. All records from bibliographic databases, Google Scholar and reference screening were imported into Covidence, where duplicates were automatically removed before blinded title and abstract screening. Only English-language studies were included (Table 1).

**Table 1.** Full search strings used in primary bibliographic databases

Database	Search string
PubMed	(‘Antimicrobial Cationic Peptides’ [MeSH] OR ‘Peptidomimetics’ [MeSH] OR ‘antimicrobial peptide*’ OR ‘host defense peptide*’ OR ‘antimicrobial peptoids*’ OR ‘AMP mimic’ OR ‘cationic peptide*’) AND (‘Cytotoxicity’ [MeSH] OR haemolysis OR ‘IC50’ OR ‘CC50’ OR ‘LD50’ OR ‘selectivity index’ OR ‘therapeutic index’ OR ‘toxicity assay’) AND (‘Protein Binding’ [MeSH] OR ‘serum binding’ OR ‘drug stability’ OR ‘protease degradation’ OR bioavailability OR ‘pharmacokinetics’ [MeSH]) AND (‘drug-resistant’ OR ESKAPE)
Scopus	TITLE-ABS-KEY ((‘antimicrobial peptide*’ OR ‘AMP’ OR ‘host defense peptide*’ OR ‘cationic peptide*’ OR ‘peptidomimetic*’) AND (‘toxicity’ OR ‘cytotoxicity’ OR ‘IC50’ OR ‘CC50’ OR ‘haemolysis’ OR ‘therapeutic index’ OR ‘serum binding’ OR ‘protein binding’) AND (‘drug-resistant’ OR ‘ESKAPE’)) AND DOCTYPE (ar) AND LANGUAGE (english) AND PUBYEAR > 1999 AND PUBYEAR < 2026 AND (SUBJAREA (biochem) OR SUBJAREA (phar) OR SUBJAREA (immu) OR SUBJAREA (medi)) AND (LIMIT-TO (LANGUAGE, ‘English’)) AND (EXCLUDE (EXACTKEYWORD, ‘Unclassified Drug’) OR EXCLUDE (EXACTKEYWORD, ‘Antiinfective Agent’) OR EXCLUDE (EXACTKEYWORD, ‘Amino Acid Sequence’) OR EXCLUDE (EXACTKEYWORD, ‘Female’) OR EXCLUDE (EXACTKEYWORD, ‘Circular Dichroism’) OR EXCLUDE (EXACTKEYWORD, ‘Vancomycin’) OR EXCLUDE (EXACTKEYWORD, ‘Scanning Electron Microscopy’) OR EXCLUDE (EXACTKEYWORD, ‘Male’) OR EXCLUDE (EXACTKEYWORD, ‘Ciprofloxacin’) OR EXCLUDE (EXACTKEYWORD, ‘Ampicillin’) OR EXCLUDE (EXACTKEYWORD, ‘Antifungal Activity’) OR EXCLUDE (EXACTKEYWORD, ‘Synergistic Effect’) OR EXCLUDE (EXACTKEYWORD, ‘Cell Line, Tumor’) OR EXCLUDE (EXACTKEYWORD, ‘Reactive Oxygen Metabolite’) OR EXCLUDE (EXACTKEYWORD, ‘Rifampicin’) OR EXCLUDE (EXACTKEYWORD, ‘Mycobacterium Tuberculosis’) OR EXCLUDE (EXACTKEYWORD, ‘Erythromycin’) OR EXCLUDE (EXACTKEYWORD, ‘Antineoplastic Activity’) OR EXCLUDE (EXACTKEYWORD, ‘Antifungal Agent’))
Embase	(‘antimicrobial peptide*’ OR amp OR ‘host defense peptide*’ OR ‘cationic peptide*’ OR peptidomimetic* OR ‘AMP mimic’) AND (toxicity OR cytotoxicity OR haemolysis OR ic50 OR cc50 OR ‘therapeutic index’ OR ‘serum binding’ OR ‘protein binding’ OR bioavailability OR pharmacokinetics) AND (‘drug-resistant’ OR ESKAPE)

While the core conceptual search terms were consistent across all databases, specific search strings were adapted to each database’s syntax, controlled vocabularies (e.g. MeSH for PubMed, Emtree for Embase) and field codes (e.g. TITLE-ABS-KEY for Scopus) to maximize literature retrieval.

### Inclusion and exclusion criteria

Experimental studies of Gram-negative and Gram-positive bacteria, particularly ESKAPEE pathogens, assessing safety using mammalian cell lines [e.g. Vero, Madin–Darby canine kidney (MDCK), human corneal epithelial cells] or animal models were included. To quantify the translational barriers and the potency-safety trade-off, inclusion required reporting of quantitative safety, toxicity or physiological stability metrics [e.g. CC_50_, haemolytic dose (HD_50_), serum protein binding or *in vivo* toxicity]. Paired data were additionally required to calculate the selectivity index (SI) and map the potency-safety landscape. Studies reporting *in vitro* efficacy of AMPs were excluded. Reviews, conference abstracts, editorials, studies on non-bacterial organisms, and studies testing conventional antibiotics alone were also excluded.

### Study selection

**Figure 1.**
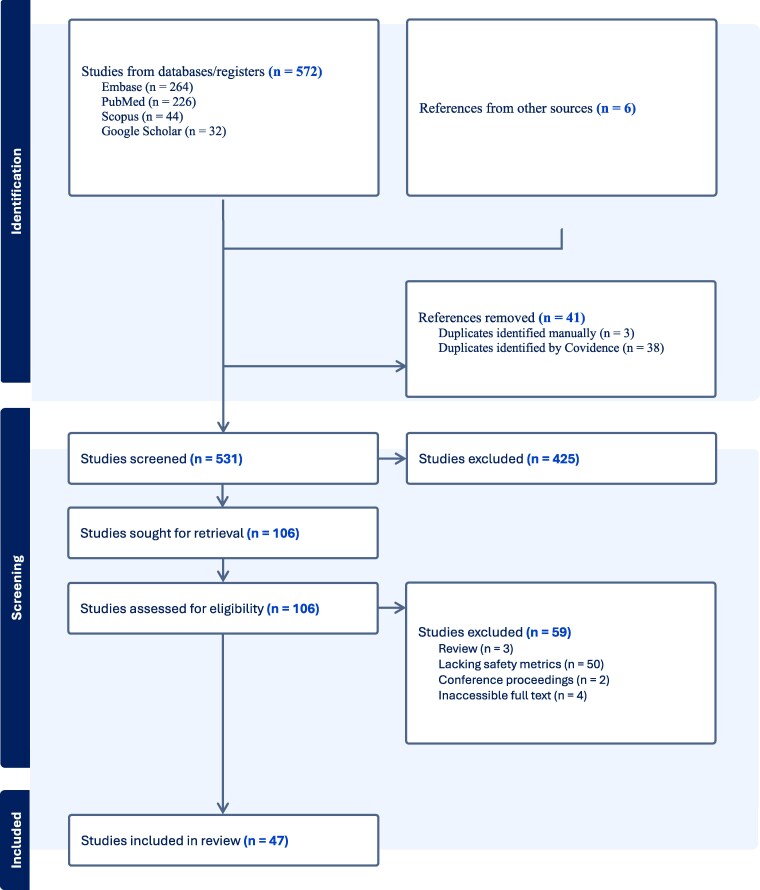
PRISMA flow diagram of study selection process for the systematic review.

The review followed a prespecified protocol registered on PROSPERO (CRD420251085424). Two reviewers (E.S.M. and Y.M.Y.) independently screened titles and abstracts, followed by full-text assessments for final inclusion. Disagreements were resolved by discussion or by a third reviewer. The selection process and flow of records are summarized according to the Preferred Reporting Items for Systematic Reviews and Meta-analyses (PRISMA) 2020 flow diagram (Figure 1).

#### Data extraction and unit harmonization

Data extraction was performed independently by two reviewers (E.S.M., Y.M.Y.) using a standardized Microsoft Excel template designed to capture details including peptide characteristics, target organism, MIC values, haemolysis and cytotoxicity profile, serum binding effect, stability data (proteases, thermal, pH, ion/salt, storage), SI and *in vivo* study outcomes. To ensure mathematical consistency and biological comparability across highly heterogeneous studies, all concentration measures for antibacterial potency and host safety outcomes were systematically standardized to micromolar (µM). This uniform molar framework is methodologically essential to eliminate the evaluation bias introduced by mass-based concentration metrics when directly comparing structurally diverse agents, particularly high-molecular-weight natural and synthetic peptides versus significantly smaller, non-natural peptidomimetics. Source values reported in mass units (e.g. µg/mL) were converted to µM using the peptide’s molecular weight according to the standard formula: µM = (µg/mL ÷ MW in g/mol) × 1000. Data reported as millimolar (mM) were converted to µM by multiplying by 1000. For studies lacking molecular weight data, molecular weight was calculated from the peptide's amino acid sequence data using the Expasy-ProtParam tool (Expasy—ProtParam). Variations introduced by chemical salts, counterions and mixtures were controlled by basing conversions on the active base peptide core. Maintaining this rigorous molar standardization across text, tables and figures ensured an accurate, molecule-for-molecule comparison of drug–target interactions and therapeutic indices.

#### Quality and risk-of-bias assessment

Data quality and risk of bias (RoB) were assessed independently by the two reviewers (E.S.M. and Y.M.Y.). For *in vivo* mammalian studies (e.g. murine or rat models), the SYRCLE's RoB tool was applied to assess baseline characteristics, allocation concealment and blinding.^33^ For *Galleria mellonella* models, only applicable bias domains (random allocation, binding of outcome assessment) were evaluated. For *in vitro* studies (e.g. MIC, haemolysis and cytotoxicity), methodological quality was assessed by clarity of the experimental design, use of appropriate controls, and standardization of bacterial inocula and cell lines.

### Statistical analysis

Given the high heterogeneity in study designs, target organisms and outcome reporting, a formal meta-analysis was not feasible. Instead, unweighted pooled geometric mean (GM), ratios and categorical summaries were used to compare potency, safety and stability across AMP classes. This quantitative framework was selected specifically to provide a standardized, descriptive synthesis of the potency-safety landscape rather than to generate precision-weighted effect estimates. To address censored MIC values, midpoint multipliers were applied. Standard antimicrobial susceptibility testing uses 2-fold serial dilution protocols, as per CLSI M07 broth microdilution principles.^34^ Within this standardized concentration gradient, censored values are mathematically bounded: right-censored values (>x) were estimated at the midpoint of the next 2-fold dilution (1.5×x) and left-censored values (<x) at the midpoint of the previous step (0.75×x). This approach prevents skewing of the pooled GM assumed extremes.

Potency (GM MICs) and SI values were calculated from log-transformed data, with censored MICs managed as described. The potency-safety relationship was visualized using log-scaled GM MIC versus GM SI plots with 95% CIs.

Safety outcomes, including haemolysis and cytotoxicity, were classified using a four-tier grading system to harmonize cross-study comparisons. Haemolysis was categorized as: negligible (≤10%), low (>10%–25%), moderate (>25%–50%) and high/severe (>50%). The stringent threshold for negligible (≤10%) and low (>10%–25%) profiles reflects the regulatory standard for blood-contacting agents, where haemolysis rates are benchmarked against known non-haemolytic reference materials (≤5%) to minimize risk of adverse interactions.^35^ The high/severe (>50%) category corresponds to a level of erythrocyte destruction widely recognized, both in research and regulatory guidance, as highly deleterious and grounds for exclusion from further therapeutic development.^36^ Cytotoxicity was analysed as percent cell viability and categorized as negligible (>90% cell viability), low (75%–90%), moderate (50%–74%) or high/severe (<50%). The transition from ‘low’ to ‘moderate’ toxicity at the ≤75% boundary is a conservative adaptation of the ISO 10993–5 standard regulatory minimum. This stringent approach provides a robust categorization necessary for quantitative synthesis.^37^

Stability metrics (serum, protease, chemical, thermal and effect of ions) were reported as medians with IQR and brief condition descriptors. *In vivo* outcomes were summarized as survival percentage versus controls, with key histological and biochemical toxicity measures. Natural AMPs were excluded from formal GM SI and potency-safety landscape calculation due to the infrequent paired cytotoxicity data (CC_50_ and MIC) required for the SI calculation. All statistical analyses were performed using R 4.3.2 (packages: *boot*, ggplot2, dplyr).

## Results

### Study selection

A total of 572 records were retrieved: 264 from Embase, 226 from PubMed, 44 from Scopus, 32 from Google Scholar and 6 from manual reference screening. Following the removal of 41 duplicates, 531 records remained for title and abstract screening. Of these, 425 records were excluded for not meeting inclusion criteria, being review articles or targeting non-bacterial organisms. The full texts of the remaining 106 articles were assessed for eligibility; 50 articles were excluded for lacking quantitative safety metrics. These studies reported antimicrobial efficacy (MIC) but didn’t provide paired safety data (e.g. CC_50_, HD_10_/HD_50_ or quantifiable *in vivo* toxicity), which were required for SI calculating and mapping the potency-safety landscape. Ultimately, 47 studies fulfilled all the inclusion criteria and were included in the quantitative analysis (Figure 1). PRISMA 2020 reporting guidelines were followed, as confirmed by the checklist in Table S1 (available as Supplementary data at *JAC* Online).

### Study characteristics

The characteristics of the 136 included compounds are summarized in Table 2. This table details the types of AMPs investigated, their primary target organism, and the specific methodologies employed for safety assessment, including the source of blood cells for haemolysis and the cell lines used for cytotoxicity assay.

**Table 2.** Characteristics of included studies

Characteristics	Category	Count	Percentage
Peptide type	Natural	20	14.7
	Synthetic	70	51.5
	Peptidomimetics	46	33.8
Target organism	Multiple organisms	87	64
	Single organism	42	30.9
	Not reported	7	5.1
Blood type for haemolysis	Human RBCs	32	23.5
	Horse RBCs	19	13.9
	Mouse RBCs	3	2.2
	Not reported	82	60.3
Cell type for cytotoxicity	Epithelial^a^	27	19.9
	Kidney^b^	18	13.2
	Liver^c^	15	11
	Fibroblast^d^	6	4.4
	Immune^e^	6	4.4
	Other^f^	3	2.2
	Not reported	61	44.9
*In vivo* study	*Galleria mellonella*, murine infection model or rat models	25	18.4

^a^Epithelial cell models included 3D human gingival epithelial cell models, human corneal epithelial cells, HaCaT keratinocytes, lung epithelial cells and Caco-2 human colorectal adenocarcinoma cells.

^b^Kidney cell models comprised HEK293 cells, kidney embryonic cells, Vero cells, immortalized pig proximal tubular epithelial cells, NRK-52E rat renal proximal tubular epithelial cells and MDCK canine renal distal tubular epithelial cells.

^c^Liver cell lines included human hepatoma cell lines and a human hepatocellular carcinoma cell line.

^d^Fibroblast models consisted of mouse embryonic fibroblast cells, human skin fibroblasts, human dermal fibroblasts and L929 fibroblasts.

^e^Immune cell models included human macrophage cells, brain microglia and RAW 264.7 murine macrophages.

^f^Other cell lines included endothelial cells, dermal microvascular endothelial cells, HT1080 human fibrosarcoma cells and SH-SY5Y human neuroblastoma cells.

#### RoB assessment of in vivo animal studies

RoB was assessed for 12 studies reporting primary *in vivo* animal data using SYR-LE's risk-of-bias tool. Full domain-level assessments and assessor notes for all included studies are provided in Table S2. The studies comprised three *Galleria mellonella* models, one mixed *G. mellonella* and murine model, seven murine models, and one rat toxicity model. Across all domains, the predominant judgement was unclear risk, reflecting widespread underreporting of methodological detail rather than evidence of systematic bias. Domain 1 (sequence generation) was unclear across all 12 studies. Domain 2 (baseline comparability) was rated low risk in seven studies in which species, strain, sex and group size were reported, but as unclear in the remaining studies. Domains 3 (allocation concealment), 6 (random outcome selection) and 7 (assessor blinding) were unclear in all 12 studies, as these methods were not described. Domains 4 and 5 (random housing and caregiver blinding) were not applicable for *Galleria mellonella*–only studies; for studies with murine or rat components, these domains were also rated unclear. Domain 8 (incomplete outcome data) was low risk across all 12 studies, as outcome data were complete for all animals. Domain 9 (selective outcome reporting) was low risk in nine studies and unclear in three due to insufficient methodological detail. Domain 10 (other sources of bias) was low risk in one study (clearly group separation, no conflicts of interest, and appropriate statistical tests) and unclear in all remaining studies. These findings reflect broader patterns in the preclinical AMP studies, where randomization, allocation concealment and blinding are rarely reported. This pervasive methodological underreporting should be carefully considered when interpreting the *in vivo* safety and efficacy data synthesized in this review.

### Peptide type distribution

A total of 136 peptide entries were analysed: 20 natural (14.7%), 70 synthetic AMPs (51.5%) and 46 peptidomimetics (33.8%). The complete list of evaluated peptides is provided in Table S3. This classification facilitated clear comparisons between peptides and structurally distinct peptidomimetics designed to mimic peptide activity.

### AMP potency

**Figure 2.**
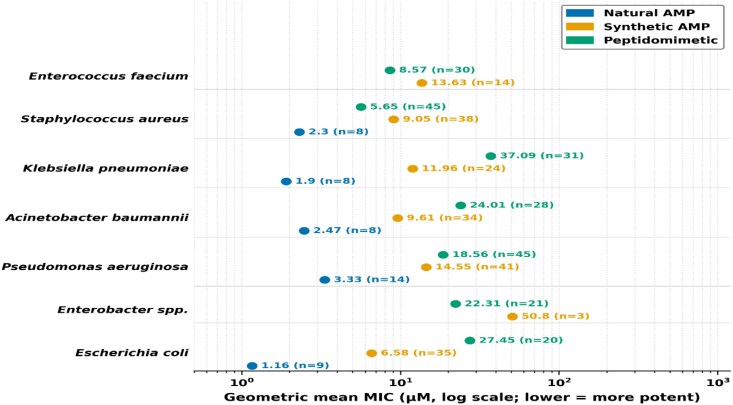
Comparison of GM MIC values (on a log scale) for natural, synthetic and peptidomimetic antimicrobial peptides (AMPs) across multiple bacterial species; lower values indicate higher potency. Point labels show GM MIC by organism for each AMP class with 95% bootstrap CIs and *n (* number of individual MIC data points). Censored MIC values were handled using the midpoint approach: ranges set to (a + b)/2; right-censored (>x) set to 1.5×x; left-censored value (<x) set to 0.75×x. *x*-axis is log_10_ (lower = more potent).

Antimicrobial efficacy, as measured by GM MICs across species, revealed a distinct potency hierarchy. Natural AMPs consistently exhibited the highest potency, requiring lower concentrations across most bacterial species compared with synthetic AMPs or peptidomimetics. This superiority was most pronounced for Gram-negative ESKAPEE pathogens. For example, against *A. baumannii*, natural AMPs had a GM MIC of 2.47 µM, approximately 4-fold lower than synthetic AMPs (9.61 µM) and more than 9-fold lower than peptidomimetics (24.01 µM). Similarly, against *E. coli*, the GM MIC for natural AMPs (1.16 µM) was substantially lower than that of synthetic AMPs (6.58 µM). Overall, the data confirmed a general potency trend of natural AMPs > synthetic AMPs > peptidomimetics for most Gram-negative species. However, this hierarchy was reversed for the Gram-positive pathogens *E. faecium and S. aureus*, in which peptidomimetics typically demonstrated higher potency (lower MIC) than synthetic AMPs (Figure 2).

### Comparative potency ratios

**Figure 3.**
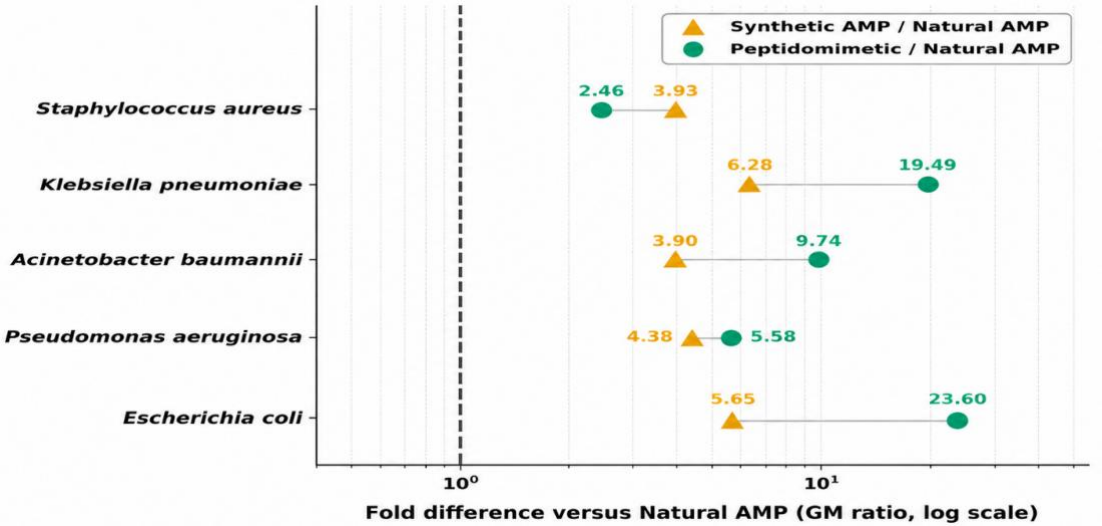
The GM MIC ratios (log scale) for synthetic and peptidomimetic antimicrobial peptides (AMPs) compared with natural peptides across different organisms for which paired natural AMP data were available values >1 indicate lower potency than natural AMPs. Each data point represent the pooled GM MIC ratio, with value labelled directly. The vertical dashed line at 1.0 denotes no difference in potency relative to natural AMPs. Censored MIC values were handled using the midpoint approach: ranges set to (rules(a+b)/2; right-censored values (>x) set to 1.5×x; left-censored value (<x) set to 0.75×x.

The ratio analysis (synthetic AMP or peptidomimetic MIC divided by natural AMP MIC) was used to normalize the data and confirm the potency differences across classes. Using the natural AMP GM MIC as the baseline, both synthetic AMPs and peptidomimetics exhibited MICs greater than 1.0 ×  the baseline. The greatest potency deficits were observed for peptidomimetics against Gram-negative bacteria, particularly against *E. coli* (23.60-fold higher) and *K. pneumoniae* (19.49-fold higher) (Figure 3). No data were available for natural AMPs against *E. faecium* and *Enterobacter* spp.

### Host toxicity

#### Haemolysis profiles of AMPs

**Figure 4.**
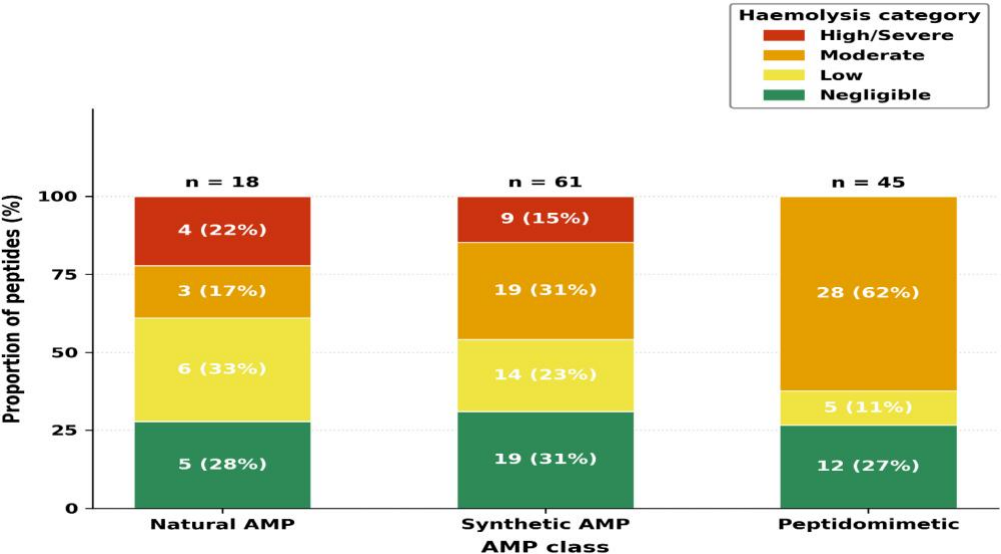
The proportions of antimicrobial peptides in each class (natural, synthetic, peptidomimetic) that fall into different haemolysis categories (negligible, low, moderate or high/severe), based on the most severe result observed per peptide. Bars show the within-class composition of unique peptides based on the highest haemolysis category observed for each peptide across all records. Categories follow the primary studies’ thresholds: negligible (≤10% RBC lysis), low (>10%–25%), moderate (>25%–50%), and high/severe (>50%). Peptides without haemolysis data were excluded from denominators. Class totals (*n*) above each bar denote the numbers of unique peptides, not assays or replicates. As assay protocols and erythrocyte sources varied (e.g. species, percentage RBCs, incubation time, readout), the percentages represent relative patterns across classes rather than harmonized absolute rates.

Haemolysis profiles for 124 peptides (natural, *n* = 18; synthetic, *n* = 61; and peptidomimetic, *n* = 45) showed distinct difference by class. Natural and synthetic AMPs exhibited broadly similar patterns of low-level toxicity, with approximately one-third of each class categorized as negligible haemolysis (28% and 31%, respectively). However, a marked difference emerged in the mid-range: synthetic AMPs shifted from low toxicity (33% in natural to 23% in synthetic) to moderate toxicity (17% in natural to 31% in synthetic). Natural AMPs had the highest proportion of high/severe toxicity (22%) compared with synthetic AMPs (15%). In contrast, peptidomimetics displayed a distinct safety profile, with no reported cases of high/severe haemolysis and most of them (62%) falling into the moderate category (Figure 4).

#### Cytotoxicity patterns by AMP class and cell line

Overall, cytotoxicity among the 124 peptides analysed was predominantly moderate (40.3%), with 29% categorized as negligible and 20.2% as low. Class-specific analysis revealed distinct safety profiles: peptidomimetics had the safest profile, with 62.2% moderate cytotoxicity and zero entries classified as highly cytotoxicity (95% CI, 0.0–7.9). Natural AMPs had the highest proportion of highly cytotoxic peptides (22.2%), while synthetic AMPs showed an intermediate distribution (14.8% high, 31.1% moderate, 31.1% negligible; Table 3).

**Table 3.** Cytotoxicity profile of AMPs across classes

AMP class	Cytotoxicity category					
	Peptides (*n*)	Negligible, *n* (%)	Low, *n* (%)	Moderate, *n* (%)	High, *n* (%)	% High (95% CI)
Natural AMPs	18	5 (27.8)	6 (33.3)	3 (16.7)	4 (22.2)	22.2% (6.4–47.6)
Synthetic AMPs	61	19 (31.1)	14 (23)	19 (31.1)	9 (14.8)	14.8% (7.0–26.2)
Peptidomimetics	45	12 (26.7)	5 (11.1)	28 (62.2)	0	0.0% (0.0–7.9)
All classes	124	36 (29)	25 (20.2)	50 (40.3)	13 (10.5)	10.5% (5.7–17.3)

Values are *n* (%) within AMP class. The percentage of high (with 95% CI) denotes the proportion of peptides rated as high cytotoxicity calculated using exact (Clopper-Pearson) binomial 95% CIs [AQ29], which are appropriate for small samples and extreme proportions. Cytotoxicity categories were harmonized as negligible, low, moderate or high; non-informative or ‘not reported’ entries were excluded. Peptidomimetics had zero high observations; precision is reflected in the 95% CIs (e.g. 0.0–7.9%).

Cell line–specific analyses revealed substantial heterogeneity and cell-context dependency, often complicated by small sample sizes. Renal cell lines were particularly highly sensitive: AMPs tested on human embryonic kidney cells exhibited responses ranging from negligible to high toxicity.^38^ Polymyxin B induced concentration- and time-dependent cytotoxicity and apoptosis in renal proximal tubular cells.^39^ Synthetic AMPs displayed pronounced context dependence, showing negligible cytotoxicity in immune-derived THP-1 microglia-like cells yet high lytic activity with the LL-37 derivative GF-17, due to increased hydrophobicity.^10^ Conversely, synthetic cathelicidin fragments FK13 and FK16 were non-toxic to human corneal epithelial cells,^40^ and the hybrid CMA3 showed minimal cytotoxicity towards keratinocytes.^28^ Some peptoids (TM1, TM9) were cytotoxic to human corneal epithelial cells at or below their MIC.^29^ While the quantitative synthesis suggests peptidomimetics have favourable comparative safety trends, class-level conclusions must be interpreted cautiously given the profound heterogeneity of preclinical evidence. Cytotoxicity profiles are highly context-dependent and vary by cell line, media and assay conditions. For example, synthetic AMPs may show negligible toxicity in some cell lines but severe toxicity in others. Therefore, the SI findings in this review represent general estimates of therapeutic windows based on currently available data, rather than absolute pharmacological rules. Given this profound context dependency, the overall class dominance must be interpreted cautiously for clinical applicability.

### Selectivity index and potency-safety landscape

Analysis of selectivity and the potency-safety relationships revealed a critical therapeutic trade-off that was highly organism specific. The trade-off refers to the observations that agents with the highest antimicrobial activity were often not the safest when cytotoxicity was considered. The SI, calculated as the ratio of GM CC_50_ to GM MIC, was used as the primary metric; a higher value indicates a greater safety margin. By this metric, peptidomimetics were frequently the safest class tested against pathogens. This superior safety profile was most apparent for the Gram-positive bacteria (*E. faecium*), where peptidomimetics had a substantially higher GM SI (28.03) compared with synthetic AMPs (10.82). Peptidomimetics also demonstrated a superior safety profile against most Gram-negative species, including *P. aeruginosa* (GM SI 8.12 versus 1.71 for synthetic AMPs).

**Figure 5.**
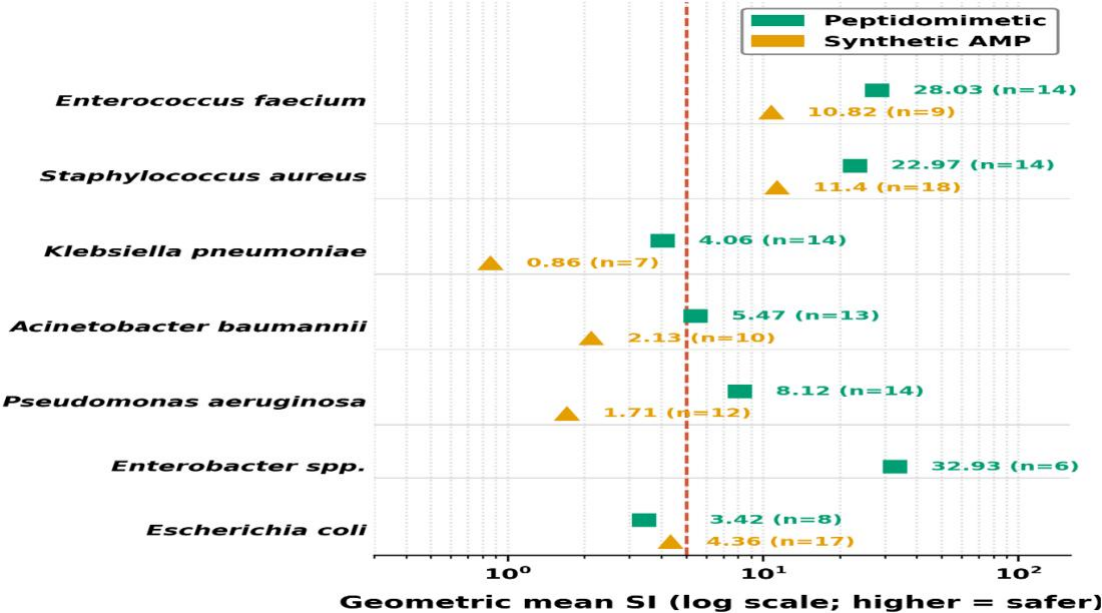
GM SI values (on a log scale) for synthetic AMPs and peptidomimetics across various organisms; higher SI values indicate greater safety margins for the peptides. Each data point is labelled with GM SI and sample size (n). A vertical dahed line at SI= 5 marks the minimum threshold considered indicative of acceptable selectivity.

Of the 20 natural AMP entries, 13 had MIC and CC_50_ values reported, while 7 lacked one or both values. However, unlike synthetic AMPs and peptidomimetics where SI values were either directly reported or could be manually calculated from paired MIC and CC_50_ data, none of the natural AMP entries provided a calculable organism-specific SI. This was because the CC_50_ and MIC values in natural AMP studies were derived from different experimental conditions or separate studies, precluding valid SI calculation. Consequently, all 20 natural AMP entries were excluded from the formal GM SI and potency-safety landscape analysis. This exclusion reflects a reporting limitation in the natural AMP literature rather than an inherent characteristic of the agents themselves, hence the comparative SI findings for synthetic AMPs and peptidomimetics should be interpreted with this constraint in mind (Figure 5).

**Figure 6.**
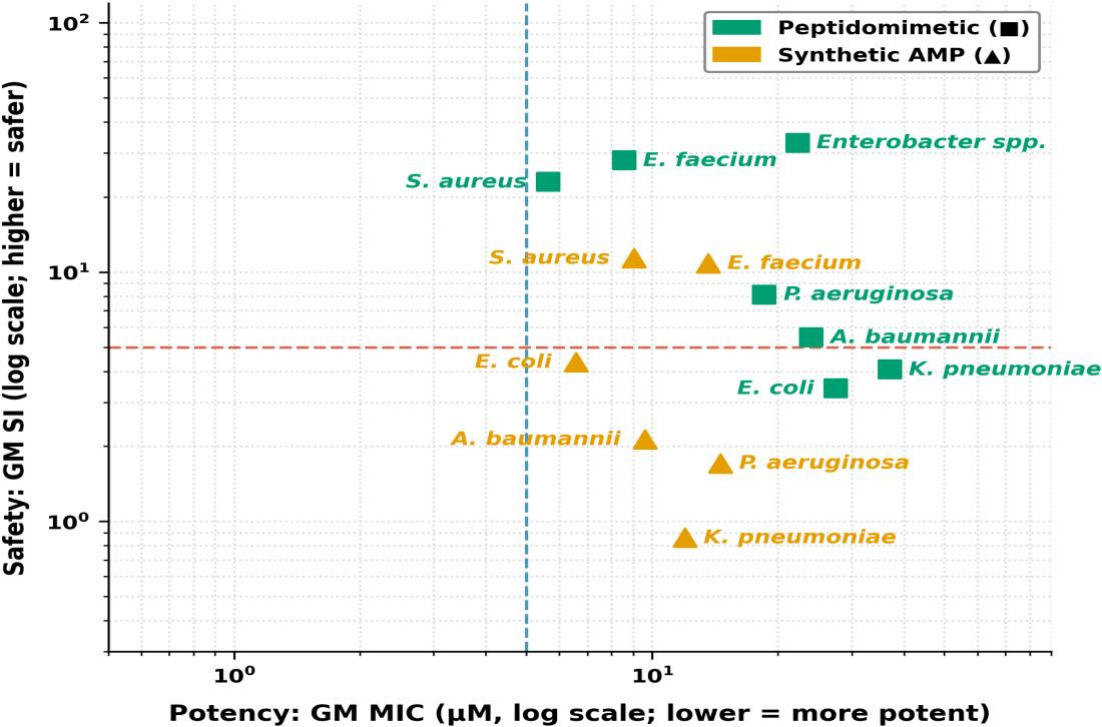
Scatter plot of organism-specific GM values under midpoint parsing rules, restricted to organism-class pairs with both MICs and SI data. The *x*-axis shows GM MIC (µM, log scale; lower = more potent) and the *y*-axis shows GM SI (log scale; higher = more selective). A vertical blue dahed line at MIC = 5 µM and a hortizontal red line at SI = 5 delineate the the upper-left quadrant, which highlights the most favourable potency-safety region.

The potency-safety landscape provides a visual summary of the efficacy-versus-safety relationship for synthetic AMPs and peptidomimetics. This analysis, which plots GM MIC (potency) against GM SI (safety), revealed nuanced, organism-specific trade-offs between the two synthetic classes (natural AMPs were excluded due to insufficient paired data; Figure 6). The analysis confirmed that while synthetic AMPs generally hold an advantage in raw potency (lower MIC), this was often offset by the superior safety margin of peptidomimetics. For example, peptidomimetics were superior in both dimensions against *E. faecium*, with a lower MIC and substantially higher SI (MIC 8.57 µM versus 13.6 µM; SI 28.0 versus 10.8 for synthetics). The most significant trade-offs were observed against Gram-negative pathogens such as *P. aeruginosa* and *K. pneumoniae*, where synthetic AMPs maintained higher potency but peptidomimetics provided a substantially greater host-safety margin (SI up to 8.12 for *P. aeruginosa*). Overall, the landscape confirmed that the most favourable profiles depended on the pathogen, necessitating organism-specific decisions to balance the efficacy of synthetic AMPs against the reduced toxicity of peptidomimetics (Figure 6).

### Serum binding effect

Investigation into the serum binding properties of various natural AMPs revealed consistently high affinity to plasma components. Binding ranged from 63% (polyphemusin-1) to 92% (thanatin) as measured by ultrafiltration in 100% human plasma.^38^ This high degree of protein binding of natural peptides was comparable to that observed for the clinical standard, polymyxin B (92%–99%).^41^ These findings suggest that a substantial proportion of these natural therapeutic candidates would be highly protein-bound in systemic circulation, potentially limiting their free *in vivo* free concentration and bioavailability.^42^

### Biophysical stability

#### Serum and plasma stability

The stability of peptides in biological fluids such as serum and plasma reflects their resistance to chemical degradation, a critical determinant of half-life and therapeutic potential. The principal mechanism affecting stability in these fluids is enzymatic degradation by proteases. The current analysis highlighted that chemical modifications can substantially improve stability: for example, the D-forms of a synthetic peptide, pepdD2, showed a more than 12-fold increase in half-life compared with its parent peptide, pepD2.^43^ In contrast, some synthetic peptides were rapidly degraded; BP607 was the least stable peptide, undergoing complete degradation after just 10 min in human serum. Residual peptide concentration varied: BP76 retained only 15% after 60 min in 25% (v/v) human serum, while BP145 retained 35%.^44^ Furthermore, the antimicrobial activity of the synthetic peptide LI14 was also negatively affected, with MIC values increasing by 4- to 8-fold in the presence of 10% serum, indicating that binding to serum components reduces the available free drug concentration.^27^

#### Protease stability

Protease stability was a strongly dependent on chemical modification.^45^ The D-amino acid peptide pepdD2, consistent with the stereospecificity of proteolytic mechanisms, was not digested by proteases.^43^ Sequence-specific effects were observed for the synthetic peptide LI14, which maintained full activity after incubation with pepsin but lost activity after trypsin and papain digestion.^27^ A significant finding for peptidomimetics (TM4, TM5, TM14, TM18 and TM19) demonstrated inherent resistance to proteolytic cleavage, as their MICs remained unchanged after exposure to proteinase K and trypsin.^46^

#### Thermal stability

Thermal stability is a critical consideration for the successful clinical transition of AMPs, affecting long-term storage and viability following sterilization. The synthetic peptide LI14 was stable between 40°C and 100°C, but lost its antibacterial activity by 4-fold at 121°C.^27^ The synthetic AMP, SAMP-A4, remained stable for 1 h at temperatures up to 90°C.^47^ In contrast, melimine retained its activity following sterilization at 121°C for 15 min.^48^ Furthermore, polymyxin B exhibited thermal stability up to 70°C.^39^

#### pH stability

The stability of antimicrobial agents across varying pH levels is crucial for determining their therapeutic utility in diverse biological environments, such as wound sites or the gastrointestinal tract. The synthetic peptide LI14 was stable and active across a broad pH range from 2 to 12.^27^ Similarly, melimine remained essentially unchanged under both acidic (pH 4.5) and basic (pH 9.4) conditions.^48^

However, for some compounds, even minor pH variations significantly affected functional efficacy. For instance, the antimicrobial activity of the peptoid TM1 was sensitive to acidification, resulting in 2-fold reduction in potency.^49^

#### Ion/salt stability

The natural AMP brevinin-1pl, and synthetic peptides Ala16-[Lys4]brevinin-1pl,^26^ LI14 and TS lost activity in the presence of divalent cations.^19,27^ Conversely, the hybrid peptide PA13 showed a nuanced profile of salt sensitivity. It maintained stable antimicrobial activity against *P. aeruginosa* in the presence of NH_4_^+^, Zn^2+^, Fe^3+^ and Ca^2+^ ions, but exhibited a marked decrease in activity when exposed to Mg^2+^ ions, and its MIC increased 2-fold in the presence of K^+^.^21^ Notably, the modified analogue, des-Ala16-[Lys4]brevinin-1pl, exhibited enhanced tolerance to Na^+^ compared with the parent peptide brevinin-1pl.^26^

### In vivo efficacy and safety

#### Galleria mellonella model

The successful outcomes observed in *in vivo* infection models, particularly employing *Galleria mellonella* larvae as a proxy for mammalian toxicity and efficacy, confirm that rational design can overcome critical barriers such as toxicity and stability, leading to improved therapeutic candidates.^1,26^

Comparative safety data for the novel synthetic peptides BP76 and BP145 demonstrated favourable safety outcomes in this model when compared with the conventional lipopeptide antibiotic colistin (polymyxin E). At a high dosage of 50 µg/larva, BP76 led to 100% survival, and BP145 achieved 70% survival within 24 h. In contrast, colistin at the same dose caused 50% mortality. These positive safety results may be attributable to peptide degradation *in vivo*: BP76 was the least stable peptide in human serum, undergoing complete degradation after just 10 min, with only a trace remaining after 60 min; BP145 showed slightly greater stability after 60 min.^44^

Similarly, the synthetic peptide LI14 demonstrated strong therapeutic efficacy. A high dose of LI14 (50 mg/kg) resulted in 100% survival in larvae infected with MRSA T144 and 75% survival in those infected with *E. coli* B2. This robust efficacy contrasts with the infected vehicle control group, which experienced 100% mortality within 48–72 h post-infection. This safety may be related to LI14’s serum binding and/or protease-mediated degradation by trypsin and papain.^27^

Promising results were found with phage-encoded AMPs. The efficacy of PE04-1, PE04-1(NH2) and PE04-2 correlated with their low cytotoxicity profile. *In vitro* assays using human hepatoma cell lines showed that peptide concentrations at the MIC level did not affect cell viability. This inherent safety translated into strong *in vivo* tolerability: control larvae injected with the peptides alone maintained survival throughout the observation period, confirming that the treatments were not inherently toxic to *Galleria mellonella* at the MIC levels. Crucially, when administered at their respective MICs against XDR *A. baumannii*, these peptides enhanced survival rates in *Galleria mellonella* (Day 10 after infection). The high lethality of the infection model was confirmed by the rapid decline of untreated larvae infected with *A. baumannii* ATCC19606, which showed less than full survival at Day 5.^50^

Structural modification has also proved effective in enhancing *in vivo* protection by addressing the problem of non-specific lytic activity. The analogue des-Ala16-[Lys4]brevinin-1pl was rationally designed to reduce the hydrophobicity of its parent peptide (brevinin-1pl), a strategy to mitigate strong haemolytic activity and improve selectivity.^26^ This modification successfully enhanced selectivity, yielding a therapeutic index (TI) value of 55.77–80.63 against Gram-negative bacteria. This optimized analogue markedly improved survival outcomes against drug-resistant *E. coli* in the *Galleria mellonella* model. In larvae infected with the colistin-resistant *E. coli* 13846, survival improved from 30% (untreated) to approximately ≥80% following treatment. Similarly, in larvae infected with the carbapenem-resistant *E. coli* 2340, survival rates increased from 50% to 90%.^26^

#### Murine infection models

The synthetic cathelicidin-based peptide GW18 demonstrated excellent systemic safety, causing no acute toxicity over 72 h following a single IV dose (20–40 mg/kg), and effectively suppressed the dissemination of MRSA from the peritoneal infection site to the target organs, including the blood and lung tissues.^51^ Similarly, the synthetic peptide LI14 also showed strong systemic tolerability: daily intraperitoneal dosing at 10 mg/kg for 6 days did not result in changes in body weight, blood cell profiles, serum biochemistry or the histology of major organs (heart, liver, spleen, lung and kidney). This safety profile supported robust therapeutic activity in a neutropenic mouse thigh infection model against MRSA T144 and *E. coli* B2, where LI14 significantly reduced bacterial loads by approximately 2 log_10_ at 10 mg/kg and 4 log_10_ at 20 mg/kg.^27^

The frequent conflict between projected *in vitro* toxicity and actual host safety reveals an *in vivo* toxicity paradox for certain AMPs. The amphibian-derived peptide PPV1 exemplifies this: despite exhibiting moderate haemolytic action on horse erythrocytes *in vitro*, it had low systemic toxicity *in vivo* in mice. The survival rate of CD-1 mice remained the same as in the control group for up to 8 days after twice-daily intraperitoneal injection (5 μg/g), and histological examination confirmed no obvious morphological changes in kidney tissue. This *in vivo* selectivity enabled successful therapeutic use, significantly improving survival in *S. aureus*–infected mice compared with treatment with vancomycin.^30^ Similarly, the hybrid AMP CMA3 demonstrated potent anti-endotoxin and anti-inflammatory activity in a septic shock model (LPS *E. coli* O111:B4), significantly enhancing mouse survival by over 50% at 40 h and significantly reducing WBC count and serum nitric oxide level following intraperitoneal administration at 0.5–1 mg/kg.^28^

Conversely, the highly amphipathic LL-37 analogue GF-17 exhibited marked acute toxicity in a mouse acute lung injury model following intranasal inoculation (2.4 mmol/L). This treatment resulted in an average body weight loss of 2.3–2.4 g over 24 h and provoked a strong local inflammatory response, characterized by a 3.32-fold increase in protein and a 4.66-fold increase in total cell counts in the bronchoalveolar lavage fluid. Histological analysis confirmed severe pulmonary interstitial oedema and neutrophil infiltration in GF-17-treated lungs, underscoring that host safety remains highly context and route dependent.^10^

Peptidomimetics have also demonstrated significant and multifaceted activity in localized infection models. Peptoid-1, administered intraperitoneally at 4 mg/kg, achieved a substantial 2 log-order reduction in *S. aureus* peritoneal bacterial counts and reduced mortality by 75% in a murine infection model, while maintaining an absence of overt toxicity, with mice remaining healthy and active.^52^ Additionally, the protease-resistant peptidomimetic P13#1 provided both antimicrobial and anti-inflammatory synergy in a localized subcutaneous *S. aureus* air pouch model. It reduced bacterial cfu counts by approximately 50-fold compared with controls, without apparent toxicity to the host at the effective dose. Furthermore, it significantly inhibited pro-inflammatory cytokine release (NO, IL-6, TNF).^53^

#### Toxicity in rat models

Rat models can be used to characterize systemic drug safety, pharmacokinetics and mechanisms of dose-limiting toxicity for antimicrobial agents.^54^ Systemic administration of polymyxin B in Wistar rats (4 mg/kg/day intraperitoneally for 5 days) produced a classic, severe nephrotoxicity profile consistent with acute kidney injury. Functionally, the treatment significantly elevated serum creatinine with a corresponding decrease in creatinine clearance. Additionally, the drug severely compromised renal haemodynamics, resulting in reduced renal blood flow and a marked increase in renal vascular resistance. At the cellular and ultrastructural level, extensive vacuolization, organelle disruption, and the presence of swollen and disorganized mitochondria were observed in the proximal tubular epithelial cells. Mitochondrial dysfunction was confirmed by the release of cytochrome into the cytosol and the presence of massive podocyte lesions in the glomeruli. Histopathological analysis confirmed widespread tubulointerstitial injury, including renal cortical tubular necrosis, diffuse interstitial inflammation, interstitial oedema, cellular flattening with tubular dilatation, and focal areas of denuded basement membrane.^39^

## Discussion

Antimicrobial peptides and peptidomimetics continue to attract significant attention as potential alternatives to conventional antibiotics, particularly against MDR pathogens.^43^ This systematic review of 136 peptide entries established a consistent GM MIC hierarchy: natural AMPs exhibited the highest overall potency across ESKAPEE pathogens. This intrinsic potency likely reflects their highly optimized, nature-derived, membrane-targeting mechanisms.^21^ On the other hand, peptidomimetics demonstrated greater potency against Gram-positive pathogens, including MDR *S. aureus* compared with the synthetic AMPs.^46^ Potency is closely linked to physicochemical properties such as net positive charge, amphipathicity and secondary structure, which underpin their membrane-disrupting activity.^14,48^ The structural determinants of AMP efficacy are multifactorial but centre on membrane interaction. Cationicity and electrostatic interaction are paramount for the initial selective interaction with highly anionic microbial membranes, which are rich in LPS in Gram-negative bacteria or in lipoteichoic acids in Gram-positive cells.^10^ Equally critical is the balance of amphipathicity and hydrophobicity: high amphipathicity strongly correlates with activity, yet excessive hydrophobicity, often resulting from lipidation or targeted substitutions, may compromise selectivity by increasing non-specific interactions with zwitterionic host membranes, thereby raising haemolytic and cytotoxic risk.^38,43^

The clinical viability of AMPs for systemic application remains fundamentally limited by a persistent translational barrier, where superior *in vitro* potency frequently fails to translate into reliable *in vivo* efficacy.^31^ Achieving high potency while maintaining selectivity requires precise tuning of the AMPs’ physicochemical properties. Failures arise when enhanced potency is accompanied by increased host toxicity: for example, the synthetic LL-37 derivative, GF-17, with greater amphipathicity and hydrophobicity, exhibited higher haemolytic activity.^10^ This *in vitro* lytic risk translated directly *in vivo*, resulting in a sharp decrease in WBC and neutrophil counts and inducing acute lung injury in mice, with notable body weight loss within 24 h. Moreover, the non-selective membrane-disrupting mechanism inherent to cationic AMPs introduces significant host cell toxicity.^10^ This challenge is exacerbated in sensitive tissues such as the kidney, where polymyxin B accumulates and damages renal proximal tubular cells.^39^

The successful systemic activity of some AMPs, despite *in vitro* instability, presents a compelling pharmacological paradox. This suggests that optimized structures can circumvent plasma protein binding (PPB) and salt inhibition, the two primary causes of AMP inactivation in physiological fluids.^55^ For example, the cytotoxicity of arenicin-1 was significantly reduced in the presence of FBS due to high PPB, confirming that serum components sequester the unbound active fraction.^38,56^ Likewise, the chemically synthesized PPV1 showed moderate haemolytic activity *in vitro* but did not induce hepatic or renal toxicity in mice. This paradox highlights that *in vivo* selectivity can often exceed the limited TI predicted by simplified *in vitro* assays, suggesting that a complex physiological environment, delivery route or serum components can mitigate structural toxicity.^30^ It may be that the relatively short *in vivo* half-lives of AMPs contribute to their safety profile.

To address the fundamental instability of L-peptides, two primary structural strategies have emerged. First, D-amino acid substitution confers resistance to protease digestion by circumventing enzyme stereospecificity. This modification yields quantifiable pharmacokinetic benefits; for example, pepdD2 achieved a more than 12-fold extension of its half-life in rat plasma.^43^ However, this stability strategy introduces a critical toxicity trade-off: the prolonged host cell exposure due to enhanced biostability directly increases long-term cytotoxicity risk as evidenced by pepdD2, which became the most toxic peptide after 24 h of exposure in HEK293 cells.^43^ Second, peptidomimetics, such as peptoids, overcome the proteolytic barrier through their N-substituted glycine backbone, which confers intrinsic resistance to proteolytic degradation. Peptoid analogues such as MG06 and MG09 showed no proteolysis even after 24 h in trypsin while retaining low cytotoxicity.^57^

Furthermore, AMP activity is highly sensitive to electrostatic masking by physiological salts. Divalent cations such as Mg^2+^ and Na^+^ can significantly compromise AMP activity. While the peptide PA-13 showed resistance to many cations, its efficacy against *P. aeruginosa* was markedly decreased in the presence of Mg^2+^ and Na^+^.^21^

Although the available literature on AMP drug delivery remains limited, promising advances in novel formulations are emerging. Liposomal encapsulation of AMPs such as AMP2-Lipo-1 and AMP3-Lipo-2 significantly enhanced their activity against MRSA. Importantly, these formulations could target intracellular MRSA within HEK-293 cells, achieving lower cfu counts than vancomycin alone.^18^ This nanotechnology-based approach demonstrated both cytocompatibility and haemocompatibility, supporting its potential for IV administration. Such delivery strategies represent a critical direction for the clinical translation of AMPs, offering improved efficacy, reduced host toxicity and enhanced therapeutic applicability.^18^

Despite extensive preclinical research, the clinical pipeline for AMPs reflects the immense difficulty of balancing antibacterial potency with host safety and pharmacokinetics. Currently, several AMPs are in various stages of preclinical or clinical development; however, systemic administration is severely constrained by pharmacokinetic/pharmacodynamic (PK/PD) hurdles.^10,26^ As observed in our analysis, AMPs are highly susceptible to rapid proteolytic degradation and can exhibit extraordinarily high serum protein binding, as exemplified by the 92% to 99% binding observed for polymyxin B.^41^ This extensive protein binding drastically reduces the free, unbound drug fraction in systemic circulation, making it exceptionally difficult to maintain *in vivo* exposure above the MIC without reaching toxic concentrations.^42^ To overcome these limitations, future delivery strategies are increasingly focusing on encapsulating AMPs within liposomes, hydrogels or nanoparticles to improve their bioavailability and half-life.^58^

Beyond PK/PD constraints, commercial viability and regulatory approval are hampered by the high cost and complexity of solid-phase peptide synthesis for long natural peptides. This highlights the economic necessity of engineering shorter peptidomimetics or truncated analogues to reduce manufacturing costs without losing efficacy.^43,57^ Furthermore, while AMPs generally have a lower risk of resistance emergence than traditional antibiotics due to their targeting the evolutionarily conserved cell membrane, bacteria can still develop adaptive resistance mechanisms. A primary example is the bacterial modification of LPS, such as the addition of 4-amino-L-arabinose to lipid A. This modification decreases the net negative charge of the outer membrane, thereby repelling cationic AMPs and diminishing their binding affinity, as historically observed with colistin resistance.^13,59^ Therefore, for next-generation peptidomimetics to successfully advance through the translational pipeline, they must be rationally designed to overcome these economic, pharmacokinetic and adaptive resistance hurdles.

### Limitations

Despite its breadth, this review has limitations. The analysis synthesized data from 47 primary studies employing diverse methodologies and bacterial strains, and inconsistent outcome measures, which precluded direct cross-study comparisons. Variability in the reporting of preclinical parameters such as PPB, stability under physiological conditions, and detailed protease degradation profiles significantly constrained robust quantitative synthesis, necessitating cautious interpretation of therapeutic indices.

A primary limitation of our quantitative synthesis is its reliance on an unweighted pooled GM approach. Because formal meta-analytical weighting was precluded by profound study-level heterogeneity, large comprehensive studies and small single-peptide studies contribute equally to the pooled GM calculation. Readers should therefore interpret these pooled GM estimates strictly as descriptive summaries of the currently available evidence, highlighting comparative trends rather than as definitive, precision-weighted effect estimates.

### Conclusions

The clinical viability of AMPs is dictated by a strict potency-safety trade-off against ESKAPEE pathogens. While natural AMPs demonstrate potent *in vitro* activity, their systemic application is significantly constrained by dose-limiting host cytotoxicity (such as substantial haemolysis and mammalian cell toxicity), rapid degradation and low bioavailability. Conversely, within the constraints of the available heterogeneous data, rationally designed peptidomimetics exhibit a more favourable comparative safety profile, exhibiting negligible to moderate cytotoxic effects across diverse cell lines, and enhanced proteolytic stability. These features along with higher SIs against several key pathogens, highlight the promise of peptidomimetics, whose targeted chemical engineering offers a viable pathway to optimize the balance between antimicrobial efficacy and host safety. Given the substantial methodological variability across preclinical studies, these trends represent comparative advantages rather than definitive class-level superiority. Importantly, natural AMPs were excluded from the SI analysis due to the absence of paired, same-study MIC and cytotoxicity data; the comparative SI findings for synthetic AMPs and peptidomimetics should therefore be interpreted within this specific reporting constraint.

However, realizing the full therapeutic potential of these agents requires moving beyond standard *in vitro* screening to address complex PK/PD barriers. Profound serum protein binding, rapid proteolytic degradation, and dose-limiting nephrotoxicity critically disrupt the free drug exposure required for *in vivo* efficacy. Future development must therefore prioritize advanced formulation strategies. Utilizing targeted nanocarrier delivery systems to physically shield these agents will be essential to optimize target site exposure, mitigate host toxicity, and enable effective systemic therapeutics against MDR infections.

### Recommendations

To accelerate the successful clinical translation of engineered AMPs and realize their potential as next-generation antibiotics, future research must adopt a strategic, multi-staged approach that addresses fundamental gaps in their delivery, *in vivo* behaviour and long-term safety. Focusing on the following research priorities will guide the crucial transition from the laboratory bench to the bedside:

*1. Advanced formulation strategies to optimize delivery*

Addressing the inherent instability and poor bioavailability of AMPs in systemic environments is the crucial first step. Research prioritizes systematic investigation of innovative delivery systems such as encapsulation in nanoparticles, liposomes, micelles or exosomes to overcome rapid enzymatic degradation, non-specific binding and short plasma half-lives, thereby maximizing the therapeutic index and enabling effective systemic dosing.

*2. Systematic preclinical validation*

Rigorous preclinical validation is essential to bridge the gap between *in vitro* promise and *in vivo* reality, which is critical for supporting subsequent clinical trials.

*3. Comprehensive safety profiling*

Prior to human testing, the long-term host interaction and safety profile of the engineered AMPs must be fully elucidated. Research must incorporate comprehensive toxicity evaluation, assessment of long-term stability under physiological conditions, and investigation of potential off-target effects or immunomodulatory potential. This thorough safety profiling will ensure that the compound is both microbiologically effective and clinically safe over the required treatment duration, thereby shortening the transition to clinical trials.

## Supplementary Material

dkag255_Supplementary_Data

## Data Availability

The data supporting the findings of this systematic review are included within the article and its Supplementary Materials. The raw data extracted from the primary studies analysed during this review are available from the corresponding author upon reasonable request. This systematic review protocol was prospectively registered with PROSPERO (registration number: CRD420251085424) on 2 July 2025.

## Supplementary data

Tables S1-S3 are available as Supplementary data at *JAC* Online.
