# Supplementary material for: Therapeutic potential and safety challenges of antimicrobial peptides and peptidomimetics against ESKAPEE pathogens: a systematic review with quantitative analysis
Source: J Antimicrob Chemother. 2026 Jul 27;81(8):dkag255. doi: 10.1093/jac/dkag255 (PMC13403570; doi:10.1093/jac/dkag255)
Supplement: dkag255_Supplementary_Data [file dkag255_supplementary_data.docx]

# Supplementary Files

## Supplementary File I. PRISMA 2020 checklist for systematic reviews.

PRISMA 2020 Checklist

**Manuscript:** Therapeutic potential and safety challenges of antimicrobial peptides and peptidomimetics against ESKAPEE Pathogens: A Systematic Review and quantitative analysis **PROSPERO Registration:** CRD420251085424 **Date:** December 2025

| Section/Topic | Item # | Checklist Item | Location in Manuscript |
| --- | --- | --- | --- |
| TITLE | | | |
| Title | 1 | Identify the report as a systematic review | Title page |
| ABSTRACT | | | |
| Abstract | 2 | See PRISMA 2020 for Abstracts checklist | Abstract section |
| INTRODUCTION | | | |
| Rationale | 3 | Describe rationale in context of existing knowledge | Introduction |
| Objectives | 4 | Provide explicit statement of objectives/questions | Introduction (final paragraph) |
| METHODS | | | |
| Eligibility criteria | 5 | Specify inclusion/exclusion criteria | Methodology: Study Selection |
| Information sources | 6 | Specify all databases, dates searched | Methodology: Search Strategy |
| Search strategy | 7 | Present full search strategies | Methodology: Table 1 |
| Selection process | 8 | Specify methods for study inclusion decisions | Methodology: Study Selection |
| Data collection process | 9 | Specify methods to collect data from reports | Methodology: Data Extraction |
| Data items | 10a | List and define all outcomes sought | Methodology: Data Extraction |
|  | 10b | List and define all other variables sought | Methodology: Data Extraction |
| Study risk of bias | 11 | Specify methods to assess risk of bias | Methodology: Quality and RoB |
| Effect measures | 12 | Specify effect measures used | Methodology: Statistical Analysis |
| Synthesis methods | 13a | Describe processes for study eligibility | Methodology: Statistical Analysis |
|  | 13b | Describe methods to prepare data | Methodology: Statistical Analysis |
|  | 13c | Describe methods to display results | Methodology: Statistical Analysis |
|  | 13d | Describe synthesis methods and rationale | Methodology: Statistical Analysis |
|  | 13e | Describe methods to explore heterogeneity | N/A - no meta-analysis |
|  | 13f | Describe sensitivity analyses | Methodology: midpoint rules |
| Reporting bias | 14 | Methods to assess risk of missing results | Limitations section |
| Certainty assessment | 15 | Methods to assess certainty in evidence | Methodology: Quality Assessment |
| RESULTS | | | |
| Study selection | 16a | Describe search results with flow diagram | Results + Figure 1 (PRISMA) |
|  | 16b | Cite excluded studies with reasons | Limitations section* |
| Study characteristics | 17 | Cite studies and present characteristics | Results: Table 2 |
| Risk of bias | 18 | Present risk of bias assessments | Methodology + Suppl. File 1 |
| Results of individual studies | 19 | Present summary statistics for each study | Results: Tables, Figures 2-6 |
| Results of syntheses | 20a | Summarize characteristics and risk of bias | Results: multiple subsections |
|  | 20b | Present results of statistical syntheses | Results: GM MIC, SI, figures |
|  | 20c | Present investigations of heterogeneity | N/A - descriptive synthesis |
|  | 20d | Present sensitivity analyses | Results: Figures 2-3 |
| Reporting biases | 21 | Present risk of bias from missing results | Limitations section* |
| Certainty of evidence | 22 | Present certainty assessments | Results/Discussion |
| DISCUSSION | | | |
| Discussion | 23a | Interpret results in context of other evidence | Discussion |
|  | 23b | Discuss limitations of evidence | Limitations section |
|  | 23c | Discuss limitations of review processes | Limitations section |
|  | 23d | Discuss implications for practice/policy/research | Recommendations section |
| OTHER INFORMATION | | | |
| Registration | 24a | Provide registration information | Methodology + Declarations |
|  | 24b | Indicate where protocol can be accessed | Declarations: PROSPERO |
|  | 24c | Describe amendments to protocol | No amendments made |
| Support | 25 | Describe sources of support | Declarations: Funding |
| Competing interests | 26 | Declare competing interests | Declarations: Conflicts |
| Data availability | 27 | Report what is publicly available | Declarations: Data Availability |

**Notes:**
* Items 16b and 21: Not provided as separate lists but acknowledged in Limitations section.
* Items 13e, 20c: Not applicable - no formal meta-analysis performed due to study heterogeneity.
* Overall PRISMA compliance: 93% (23/25 applicable items fully reported)

*From: Page MJ, McKenzie JE, Bossuyt PM, et al. The PRISMA 2020 statement: an updated guideline for reporting systematic reviews. BMJ 2021;372:n71.*

## Supplementary files II: SYRCLE’s risk of bias tool for animal studies

**SYRCLE’s Risk of Bias Assessment for In Vivo Animal Studies**

*Note: No composite overall risk score is calculated per SYRCLE guidelines; different bias domains carry different weights depending on the specific outcome assessed. G. mellonella studies: D4/D5 coded N/A (invertebrate; standard mammalian housing and caregiver domains not applicable).*

**Legend**

| **L** | Low risk of bias |
| --- | --- |
| **U** | Unclear risk of bias |
| **H** | High risk of bias |
| **N/A** | Not applicable: - domain irrelevant to model type (G. mellonella invertebrate models only) |
| **U*** | Unclear: - mixed-model study (G. mellonella + murine); domain coded at study level |

**Risk of Bias Summary Table**

| **Author, Year** | **Animal Model** | **D1** | **D2** | **D3** | **D4** | **D5** | **D6** | **D7** | **D8** | **D9** | **D10** |
| --- | --- | --- | --- | --- | --- | --- | --- | --- | --- | --- | --- |
| **Galleria mellonella models** | | | | | | | | | | | |
| **Alsaab et al., 2023** | G. mellonella | U | **L** | U | N/A | N/A | U | U | **L** | **L** | U |
| ***Assessor notes:*** *D4/D5 N/A: G. mellonella larvae are not housed in animal facility racks and do not receive daily caregiver handling. No infection challenge (toxicity assessment only). No randomisation sequence described; no blinding reported. D2 Low: n=10/group stated and group characteristics reported. D9 Low: all pre-specified outcomes reported. D10 Unclear.* | | | | | | | | | | | |
| **López-Siles et al., 2025** | G. mellonella | U | **L** | U | N/A | N/A | U | U | **L** | U | U |
| ***Assessor notes:*** *D4/D5 N/A: G. mellonella invertebrate model. BP76/BP145 safety comparison versus colistin. D2 Low: n =10/group standardised across experimental groups. No randomisation sequence or blinding described. D9 Unclear: insufficient reporting detail to confirm all pre-specified outcomes were reported. D10 Unclear: insufficient methodological reporting to exclude other bias sources.* | | | | | | | | | | | |
| **Rothong et al., 2024** | G. mellonella | U | U | U | N/A | N/A | U | U | **L** | **L** | U |
| ***Assessor notes):*** *D4/D5 N/A: G. mellonella invertebrate model. Phage-encoded AMPs (PE04-1, PE04-1(NH2), PE04-2) against XDR A. baumannii. No randomisation sequence or blinding described. D8 Low: survival tracked to day 10 with complete group reporting. D9 Low: reported outcomes consistent with stated antimicrobial objectives. D10 Unclear: insufficient reporting to exclude other bias sources.* | | | | | | | | | | | |
| **Mixed: Galleria mellonella + Murine model** | | | | | | | | | | | |
| **Shi et al., 2022** | G. mellonella & Mouse | U | **L** | U | U* | U* | U | U | **L** | **L** | **L** |
| ***Assessor notes:*** *D4/D5 coded Unclear at study level (U*): N/A applies only to the G. mellonella component (invertebrate); for the neutropenic CD-1 mouse thigh infection component (n=6/group), D4 and D5 are not reported and therefore Unclear. D2 Low: baseline characteristics (strain, n/group) reported for murine component. Neutropenic host limits immune context a translational limitation, not a structural bias. D10 Low: treatment groups clearly separated; no co-interventions beyond stated comparators; no declared COI; appropriate statistical tests throughout (log-rank, Mann-Whitney U); no carryover or contamination risk identified.* | | | | | | | | | | | |
| **Murine models** | | | | | | | | | | | |
| **Chi et al., 2024** | Mouse (ALI model) | U | U | U | U | U | U | U | **L** | **L** | U |
| ***Assessor notes:*** *D1: authors explicitly state "mice were randomly divided" into groups, partially addressing selection bias; however D1 remains Unclear because the sequence generation method (e.g., computer-generated numbers, coin toss) is not described a minimum requirement for Low risk under SYRCLE. Toxicity-only ALI model via intranasal inoculation; no live bacterial infection. Blinding not reported. D10 Unclear: limited methodological reporting precludes confident Low.* | | | | | | | | | | | |
| **Yuan et al., 2022** | Mouse (infection model) | U | **L** | U | U | U | U | U | **L** | **L** | U |
| ***Assessor notes:*** *D2 Low: male C57BL/6 mice aged 6–8 weeks (n=10/group); age, sex, and strain standardised across groups. GW18 cathelicidin-based peptide; murine MRSA peritoneal infection model; i.v. dose 20-40 mg/kg. No randomisation sequence, allocation concealment, or blinding described. D8 Low: survival and organ outcomes reported for all groups. D10 Unclear: insufficient reporting to exclude other bias sources.* | | | | | | | | | | | |
| **Wang et al., 2024 †** | Mouse (infection model) | U | U | U | U | U | U | U | **L** | **L** | U |
| ***Assessor notes (provisional):*** *[Provisional — verify against primary paper] Des-Ala16-[Lys4]brevinin-1pl murine infection model. Insufficient methodological detail in published report to assess most domains. Mouse strain, sample size, randomisation procedure, and blinding not confirmed. D8 Low: survival outcomes reported for all groups throughout observation period. D9 Low: outcomes consistent with stated study objectives. D10 Unclear: insufficient information to exclude other bias sources.* | | | | | | | | | | | |
| **Lee et al., 2016** | Mouse (septic shock) | U | **L** | U | U | U | U | U | **L** | **L** | U |
| ***Assessor notes:*** *D2 Low: 7-week-old male BALB/c mice (n=10/group); age, sex, and strain standardised across groups. LPS-only challenge does not fully recapitulate live bacterial sepsis a design limitation affecting translational validity, not a structural bias. Randomisation procedure and blinding not reported. D10 Unclear: cannot confirm equal procedural handling across all time points; no declared COI identified.* | | | | | | | | | | | |
| **Liu et al., 2020** | Mouse (infection model) | U | **L** | U | U | U | U | U | **L** | **L** | U |
| ***Assessor notes:*** *D2 Low: female C57BL/6J mice (n=10/group) for efficacy; CD-1 mice (n=10/group) for toxicity; age, sex, and strain standardised within each experiment. Randomisation procedure and blinding not described. Hepatic/renal endpoints provide useful multi-organ safety data. D10 Unclear: insufficient methodological reporting to exclude other bias sources; no COI identified.* | | | | | | | | | | | |
| **Czyzewski et al., 2016** | Mouse (infection model) | U | U | U | U | U | U | U | **L** | U | U |
| ***Assessor notes:*** *In vivo component embedded within broader in vitro/in silico characterisation paper. No randomisation, allocation concealment, or blinding described. D8 Low: bacterial counts and survival reported for all groups. D9 Unclear: selective reporting cannot be excluded given the multi-method paper structure. D10 Unclear: insufficient methodological reporting to exclude other bias sources.* | | | | | | | | | | | |
| **Caporale et al., 2023** | Mouse (infection model) | U | U | U | U | U | U | U | **L** | **L** | U |
| ***Assessor notes:*** *No randomisation sequence or blinding described. D8 Low: CFU counts and cytokine outcomes (NO, IL-6, TNF) reported for all experimental groups. D9 Low: reported outcomes consistent with stated anti-infective and anti-inflammatory objectives. D10 Unclear: insufficient reporting to exclude other bias sources; no COI identified.* | | | | | | | | | | | |
| **Rat model** | | | | | | | | | | | |
| **Vattimo et al., 2016** | Rat (toxicity model) | U | **L** | U | U | U | U | U | **L** | **L** | U |
| ***Assessor notes:*** *D2 Low: male Wistar rats (286 ± 12 g); body weight, sex, and strain standardised across treatment and control groups. Polymyxin B (4 mg/kg/day i.p. × 5 days); nephrotoxicity endpoints including serum creatinine, renal blood flow, histopathology, and mitochondrial ultrastructure. Randomisation procedure and blinding not described. D8 Low: all biochemical, functional, and histological endpoints fully reported. D9 Low: nephrotoxicity outcomes clearly pre-specified. D10 Unclear: equal procedural handling across time points cannot be confirmed; no COI identified.* | | | | | | | | | | | |

**Domain Rationale Notes**

The following criteria were applied consistently across all studies when assigning Low (L) versus Unclear (U) risk verdicts for each SYRCLE domain:

**D1: - Sequence generation:** Coded Low only when a specific randomisation method was explicitly named (e.g., computer-generated random numbers, random number table, coin toss). Statements such as "animals were randomly divided" without further description of the method were coded Unclear, as SYRCLE requires documentation of the generation process, not merely an assertion of randomisation.

**D2: - Baseline comparability:** Coded Low when the study explicitly reported that animals in all groups were matched on key baseline characteristics minimally species/strain, sex, age or weight, and group size (n). Coded Unclear when any of these details were absent or incompletely reported.

**D3: - Allocation concealment:** Coded Low only when a specific mechanism preventing foreknowledge of group allocation at the time of enrolment was described (e.g., sealed opaque envelopes, central randomisation service). No study in this review described such a mechanism; all were therefore coded Unclear.

**D4: - Random housing:** Coded N/A for all G. mellonella-only studies, as larvae are not housed in multi-cage facility racks where rack position could introduce systematic bias. Coded Unclear for all murine and rat studies where random or rotating cage assignment was not reported.

**D5: - Caregiver blinding:** Coded N/A for all G. mellonella-only studies, as larvae do not receive individualised daily care from human handlers. Coded Unclear for all murine and rat studies where blinding of animal handlers was not described.

**D6: - Random outcome selection:** Coded Low only when the study described random or systematic selection of animals for outcome assessment (e.g., random selection of animals for tissue harvest). No study in this review described such a procedure; all were coded Unclear.

**D7: - Assessor blinding:** Coded Low only when outcome assessors were explicitly described as blinded to group allocation during measurement or analysis. No study in this review reported assessor blinding; all were coded Unclear.

**D8: - Incomplete outcome data:** Coded Low when outcome data were available for all enrolled animals, with no unexplained losses, missing values, or selective exclusions. Coded Unclear only when data completeness could not be confirmed from the reported methods and results.

**D9: - Selective outcome reporting:** Coded Low when the outcomes reported in the results section were fully consistent with those specified in the methods section, with no apparent omission of pre-stated endpoints. Coded Unclear when insufficient methodological detail precluded a confident assessment.

**D10: - Other sources of bias:** Coded Low only when the study was free from all of the following: (i) declared or identifiable conflicts of interest; (ii) co-interventions that differed systematically between groups; (iii) contamination between experimental groups; and (iv) other identifiable design flaws. Coded Unclear when any of these elements could not be excluded from the available report.

## Supplementary files III: Complete list of 136 AMPs and peptidomimetics with key efficacy and safety parameters

| **No.** | **Reference** | **Peptide Name / Code** |
| --- | --- | --- |
| **1** | Waghu FH et al., 2018 | P1m |
| **2** | Waghu FH et al., 2018 | P1 |
| **3** | Waghu FH et al., 2018 | BMAP28(1-18) |
| **4** | Zhang R et al., 2021 | Therapeutic Scaffold (TS) |
| **5** | Vattimo et al., 2016 | Polymyxin B |
| **6** | Chi Y, et al., 2024 | LL-37 |
| **7** | Chi Y, et al., 2024 | GF-17 |
| **8** | Klubthawee N et al., 2020 | PA13 |
| **9** | Mirzaei R et al., 2023 | Melittin |
| **10** | Shi J, et al., 2022 | LI14 |
| **11** | Wang J et al., 2024 | Brevinin-1pl |
| **12** | Wang J et al., 2024 | des-Ala16-[Lys4]brevinin-1pl (also referred to as brevinin-1pl-del 16) |
| **13** | Edwards IA et al., 2016 | arenicin-3 |
| **14** | Edwards IA et al., 2016 | tachyplesin-1 |
| **15** | Edwards IA et al., 2016 | polyphemusin-1 |
| **16** | Edwards IA et al., 2016 | gomesin1 |
| **17** | Edwards IA et al., 2016 | protegrin-1 |
| **18** | Edwards IA et al., 2016 | Thanatin |
| **19** | Rothong P, et al., 2024 | PE04-1 |
| **20** | Rothong P, et al., 2024 | PE04-1(NH2) |
| **21** | Rothong P, et al., 2024 | PE04-2 |
| **22** | Jiang X, et al., 2019 | Hybrid peptide |
| **23** | Alsaggar M, et al., 2022 | HAZ |
| **24** | Majidiani H, et al., 2021 | d-leucine modified CM11 peptide |
| **25** | Datta A, et al., 2015 | VG16KRKP |
| **26** | Alsaab FM, et al., 2023 | HRZN-13 |
| **27** | Alsaab FM, et al., 2023 | HRZN-14 |
| **28** | Alsaab FM, et al., 2023 | HRZN-15 |
| **29** | Alsaab FM, et al., 2023 | HRZN-16 |
| **30** | Alsaab FM, et al., 2023 | HRZN-17 |
| **31** | Chen SP, et al., 2021 | pepD2 |
| **32** | Chen SP, et al., 2021 | pepD3 |
| **33** | Chen SP, et al., 2021 | pepI2 |
| **34** | Chen SP, et al., 2021 | pepdD2 |
| **35** | Eshtiaghi S, et al., 2021 | mCM11 (modified CM11) |
| **36** | Abodakpi H, et al., 2015 | Polymyxin B |
| **37** | Jahan I, et al., 2023 | BMAP-18 |
| **38** | Jahan I, et al., 2023 | BMAP-18-FL |
| **39** | Jin-Jiang H, et al., 2012 | K11 |
| **40** | Jin-Jiang H, et al., 2012 | S16 |
| **41** | Jin-Jiang H, et al., 2012 | CP-P |
| **42** | Jin-Jiang H, et al., 2012 | P18 |
| **43** | Ji S, et al., 2014 | Cecropin A–melittin (CAM) |
| **44** | Ji S, et al., 2014 | Cecropin A–melittin mutant (CAM-W) |
| **45** | Lee JK, et al., 2016 | CMA3 (an analogue of CA-MA) |
| **46** | Bolosov IA, et al.,2023 | Protegrin-1 (PG-1) |
| **47** | Bolosov IA, et al.,2023 | Lseganan |
| **48** | Bolosov IA, et al., 2023 | V16R |
| **49** | Liang X, et al.,2022 | MPII |
| **50** | Liang X, et al.,2022 | BMPII |
| **51** | Liang X, et al.,2022 | PMPII |
| **52** | Liu H, et al.,2015 | MBjAMP1 |
| **53** | Liu Y, et al.,2020 | Phylloseptin-PV1 (PPV1) |
| **54** | López-Siles M, et al.,2025 | BP76 |
| **55** | López-Siles M, et al.,2025 | BP145 |
| **56** | López-Siles M, et al.,2025 | BP607 |
| **57** | Maher S, et al.,2006 | Gallidermin |
| **58** | Mechkarska M, et al.,2023 | TtAP-1 |
| **59** | Mechkarska M, et al.,2023 | TtAP-2 |
| **60** | Mechkarska M, et al.,2023 | TtAP-3 |
| **61** | Yuan B, et al.,2022 | GW18 |
| **62** | Abdelraouf K, et al.,2012 | Polymyxin B |
| **63** | Abdelraouf K, et al.,2012 | Polymyxin B |
| **64** | Abdelraouf K, et al.,2012 | Polymyxin B |
| **65** | Swedan S, et al.,2019 | WLBU2 |
| **66** | Swedan S, et al.,2019 | WLBU2 |
| **67** | Raju SV, et al.,2021 | RM12 |
| **68** | Molchanova N, et al.,2019 | LBP-2 |
| **69** | Molchanova N, et al.,2019 | LBP-3 |
| **70** | Molchanova N, et al.,2019 | LBP-4 |
| **71** | Mohammed I, et al.,2019 | LL-37 |
| **72** | Mohammed I, et al.,2019 | FK-13 |
| **73** | Mohammed I, et al.,2019 | FK-16 |
| **74** | Li R, et al.,2021 | SAMP-A4 |
| **75** | Lata M, et al.,2024 | TLF15 |
| **76** | Lata M, et al.,2024 | FKL15 |
| **77** | Lata M, et al.,2024 | LIP15 |
| **78** | Lata M, et al.,2024 | SKL15 |
| **79** | Lata M, et al.,2024 | LGS15 |
| **80** | Sara M, et al.,2024 | TM1 |
| **81** | Sara M, et al.,2024 | TM1 |
| **82** | Sara M, et al.,2024 | TM4 |
| **83** | Sara M, et al.,2024 | TM4 |
| **84** | Sara M, et al.,2024 | TM5 |
| **85** | Sara M, et al.,2024 | TM5 |
| **86** | Sara M, et al.,2024 | TM9 |
| **87** | Sara M, et al.,2024 | TM9 |
| **88** | Sara M, et al.,2024 | TM14 |
| **89** | Sara M, et al.,2024 | TM14 |
| **90** | Sara M, et al.,2024 | TM18 |
| **91** | Sara M, et al.,2024 | TM18 |
| **92** | Sara M, et al.,2024 | TM19 |
| **93** | Sara M, et al.,2024 | TM19 |
| **94** | Blomstrand E, et al.,2024 | K9W4 |
| **95** | Blomstrand E, et al.,2024 | KR9W4 |
| **96** | Blomstrand E, et al.,2024 | R9W4 |
| **97** | Blomstrand E, et al.,2024 | R9W5 |
| **98** | Blomstrand E, et al.,2024 | Omiganan |
| **99** | Blomstrand E, et al.,2024 | Piscidin 1 |
| **100** | Green RM, et al., 2020 | MG01 |
| **101** | Green RM, et al., 2020 | MG02 |
| **102** | Green RM, et al., 2020 | MG03 |
| **103** | Green RM, et al., 2020 | MG04 |
| **104** | Green RM, et al., 2020 | MG05 |
| **105** | Green RM, et al., 2020 | MG06 |
| **106** | Green RM, et al., 2020 | MG07 |
| **107** | Green RM, et al., 2020 | MG08 |
| **108** | Green RM, et al., 2020 | MG09 |
| **109** | Green RM, et al., 2020 | MG10 |
| **110** | Green RM, et al., 2020 | MG11 |
| **111** | Chongsiriwatana NP, et al.,2011 | Pexiganan |
| **112** | Chongsiriwatana NP, et al.,2011 | Peptoid 1 |
| **113** | Chongsiriwatana NP, et al.,2011 | Ntridec-14mer |
| **114** | Nielsen JE, et al., 2022 | TM1 |
| **115** | Nielsen JE, et al., 2022 | TM2 |
| **116** | Nielsen JE, et al., 2022 | TM3 |
| **117** | Nielsen JE, et al., 2022 | TM4 |
| **118** | Nielsen JE, et al., 2022 | TM5 |
| **119** | Nielsen JE, et al., 2022 | TM7 |
| **120** | Nielsen JE, et al., 2022 | TM8 |
| **121** | Nielsen JE, et al., 2022 | TM9 |
| **122** | Nielsen JE, et al., 2022 | TM10 |
| **123** | Mishra, S.K., et al., 2025 | Melittin |
| **124** | Mishra, S.K., et al., 2025 | Mel4 |
| **125** | Mishra, S.K., et al., 2025 | TM1 |
| **126** | Mishra, S.K., et al., 2025 | TM2 |
| **127** | Mishra, S.K., et al., 2025 | TM4 |
| **128** | Mishra, S.K., et al., 2025 | TM6 |
| **129** | Mishra, S.K., et al., 2025 | TM8 |
| **130** | Mishra, S.K., et al., 2025 | TM12 |
| **131** | Mishra, S.K., et al., 2025 | TM14 |
| **132** | Mishra, S.K., et al., 2025 | TM15 |
| **133** | Mishra, S.K., et al., 2025 | TM19 |
| **134** | Rasul R, et al., 2010 | Melimine |
| **135** | Cafaro V, et al., 2023 | P13#1 |
| **136** | Czyzewski AM, et al., 2016 | Peptoid 1 |
